# Co-transfection of plasmid DNA and laser-generated gold nanoparticles does not disturb the bioactivity of GFP-HMGB1 fusion protein

**DOI:** 10.1186/1477-3155-7-6

**Published:** 2009-10-24

**Authors:** Svea Petersen, Jan T Soller, Siegfried Wagner, Andreas Richter, Jörn Bullerdiek, Ingo Nolte, Stephan Barcikowski, Hugo Murua Escobar

**Affiliations:** 1 Laser Zentrum Hannover e.V., Hannover, Germany; 2 Small Animal Clinic and Research Cluster of Excellence "REBIRTH", University of Veterinary Medicine, Bischofsholer Damm 15, D-30173 Hannover, Germany; 3 Centre for Human Genetics, University of Bremen, Leobener Strasse ZHG, D-28359 Bremen, Germany

## Abstract

Ultrashort pulsed laser ablation in liquids represents a powerful tool for the generation of pure gold nanoparticles (AuNPs) avoiding chemical precursors and thereby making them especially interesting for biomedical applications. However, because of their electron accepting properties, laser-generated AuNPs might affect biochemical properties of biomolecules, which often adsorb onto the nanoparticles. We investigated possible effects of such laser-generated AuNPs on biological functionality of DNA molecules. We tested four differently sized and positively charged AuNPs by incubating them with recombinant eGFP-C1-HMGB1 DNA expression plasmids that code for eGFP fusion proteins and contain the canine architectural transcription factor HMGB1. We were able to show that successfully transfected mammalian cells are still able to synthesize and process the fusion proteins. Our observations revealed that incubation of AuNP with the plasmid DNA encoding the recombinant canine HMGB1 neither prevented the mediated uptake of the vector through the plasma membrane in presence of a transfection reagent nor had any effect on the transport of the synthesized fusion proteins to the nuclei. Biological activity of the recombinant GFP-HMGB1 fusion protein appears to have not been affected either, as a strong characteristic protein accumulation in the nucleus could be observed. We also discovered that transfection efficiencies depend on the size of AuNP. In conclusion, our data indicate that laser-generated AuNPs present a good alternative to chemically synthesized nanoparticles for use in biomedical applications.

## Findings

Gold nanoparticles (AuNPs) are used widely for various biomedical applications including cell imaging [1], diagnostics [2], targeted drug delivery [3], and sensing [4]. Various methods have been established for AuNP generation. Many of these rely on several chemical reactions or gas pyrolysis, showing the risk of impurities or agglomeration [5]. Laser ablation in liquids showed to be a powerful tool with many advantages, having almost no restriction in the choice of source material and the ability of yielding highly pure colloidal particles[6-11]. These pure AuNPs are characterised by their unique surface chemistry free of surfactants, a feature unattainable by other methods [12-14]. X-ray photoelectron spectroscopy of such AuNPs revealed the presence of the oxidation states Au^+ ^and Au^3+ ^at the AuNP surface [15]. In previous studies we demonstrated that unmodified DNA oligonucleotides adsorb easily onto these positively charged nanoparticles [16,17], probably via amino- and keto-groups, which interact with the electron accepting surface of the generated AuNPs. However, these findings raised the possibility that more complex biomolecules could also be attracted and bound to such nanoparticles' surfaces, if incubated intentionally or unintentionally with colloidal laser-generated gold nanoparticles, even if no additional conjugation is envisaged. Such binding could have a strong effect on the properties of biomolecules and should be characterised with a view of their potential toxicity [18].

We therefore decided to analyse the possible effects of laser-generated AuNPs on DNA functionality. For this reason we incubated the charged particles with recombinant eGFP-C1-HMGB1 expression plasmids and subsequently transfected them into mammalian cells. As the HMGB1 protein is normally highly abundant in the cell nuclei, we were able to show that the treated expression plasmids are still functional and suitable for use as transcription matrix, because the transfected cells were still able to synthesize the fusion proteins, to process them and to transport them to their biofunctional destination. The effect of four differently sized nanoparticles on the activity of the eGFP-C1-HMGB1 plasmid was investigated by fluorescence microscopy. We additionally performed a binding assay to investigate structural effects on the plasmid due to AuNP co-incubation.

### Nanoparticle generation

**Figure 1:**
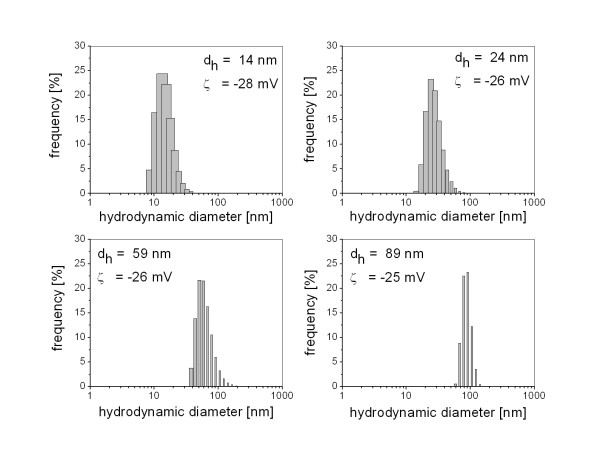
**Size distribution and surface charge of laser generated gold nanoparticles**. Gold nanoparticles (Au-NP) were generated by laser ablation in water using a femtosecond laser system (Spitfire Pro, Spectra-Physics) delivering 120 fs laser pulses at a wavelength of 800 nm (working pulse energy: 200 μJ per pulse, beam diameter: 4 mm). In order to generate four suspensions containing differently sized nanoparticles, the focal position was lowered from one generation experiment to the other (0 mm, -2 mm, -4 mm relative to the focus in air) resulting in the colloidal suspensions containing nanoparticles with mean hydrodynamic diameters of d_h _= 89 nm, d_h _= 59 nm and d_h _= 24 nm. For the generation of 14 nm sized nanoparticles, laser ablation was carried out at a focal position of -4 mm and then reirradiated for 5 min at 1 mJ with an Nd-YLF laser system (pulse length: 27 ns, 1047 nm, 5 kHz). The hydrodynamic size distribution was analysed by Dynamic Light Scattering.

AuNPs were generated by laser ablation in water, as recently reported in detail [17]. Briefly, the beam of a femtosecond laser system (Spitfire Pro, Spectra-Physics), delivering 120 fs laser pulses at a wavelength of 800 nm, was focused with a 40 mm lens on a 99.99% pure gold target placed at the bottom of a Petri dish filled with 2 mL of bidistilled water. A pulse energy of 200 μJ at 5 kHz repetition rate was employed for 12 min. According to observations of Kabashin et al. [9] the focal position was lowered from one generation experiment to the other (0 mm, -2 mm, -4 mm relative to the focus in air) in order to obtain colloidal suspensions containing AuNPs with mean hydrodynamic diameters of d_h _= 89 nm, d_h _= 59 nm and d_h _= 24 nm. The remaining small particles were removed by centrifugation at 15000 rpm for 10 min. To generate 14 nm AuNPs, laser ablation was carried out at a focal position of -4 mm, followed by a second irradiation for 5 min at 1 mJ with an Nd-YLF laser system (pulse length: 27 ns, 1047 nm, 5 kHz), as was described recently [19,20]. Characterization of nanoparticle suspensions was performed by dynamic light scattering using a Malvern Zetasizer and by UV-Vis spectroscopy using a Shimadzu 1650. The hydrodynamic number distributions and the average zeta potential of the colloids are shown in Figure 1. The zeta potential seems to be independent of the nanoparticle size, which might be explained by a similar surface charge density.

The particle mass concentration in the suspensions was determined by weighing the sediment after water evaporation.

### Au-NP and eGFP-C1-HMGB1 vector *in vitro *transfection assay

The synthesised Au-NP suspensions were sterilized by filtration through a 0.2 μm filter device (Millex-GV Sterilizing Filter Unit, Millipore, Billerica, USA). Subsequently, 250 ng of each differently sized Au-NPs were incubated for 24 h at room temperature with 1 μg of recombinant plasmid eGFP-C1-HMGB1 in a total volume of 47 μl of ddH_2_O. The time of co-incubation was intentionally kept that long as we aimed to investigate possible effects on the vector due to nanoparticle interferences. This was only possible as the circular double-stranded plasmid is not susceptible to rapid degenerative processes.

The recombinant plasmid encodes an eGFP-HMGB1 fusion protein. The HMGB1 coding sequence was derived from canine cDNA using PCR amplification (primer pair EcoR1_B15'CGGAATTCACCATGGGCAAAGGAGA3'/KpnI_B1 (5'GCGGTACCTTATTCATCATCATC-3'). The obtained PCR products were separated on a 1.5% agarose gel, recovered with QIAquick Gel Extraction Kit (QIAGEN, Hilden, Germany), cloned into the pEGFP-C1 vector plasmid (BD Bioscience Clontech) and sequenced. Twelve hours prior to transfection, 3 × 10^5 ^cells from canine mammary cell line MTH53a were seeded into 12 multi well plates. The cells were grown at 37°C and 5% CO_2 _in medium 199 (Invitrogen, Karlsruhe, Germany) supplemented with 20% FCS, penicillin, and streptomycin. For transfection, 3 μl aliquots of Fugene HD (FHD) reagent (Roche, Mannheim, Germany) were added to 47 μl of different Au-NP/eGFP-C1-HMGB1 plasmid suspensions in a total volume of 50 μl and incubated for 15 min. The three control sample sets were: (i) 1 μg of eGFP-C1-HMGB1 DNA without nanoparticles, (ii) 250 ng of Au-NPs without any plasmid DNA, and (iii) a set of Au-NPs with DNA, but without the FHD.

Following 15 min incubation at 23°C, the respective 50 μl transfection mixtures were added to cell cultures. The cells were incubated for 48 hours in medium 199 (20% FCS) at 37°C and 5% CO_2_. The uptake of plasmid DNA and expression of the eGFP-C1-HMGB1 fusion protein were verified by fluorescence microscopy. All experiments were performed in quadruples.

### Fluorescence microscopy

**Figure 2:**
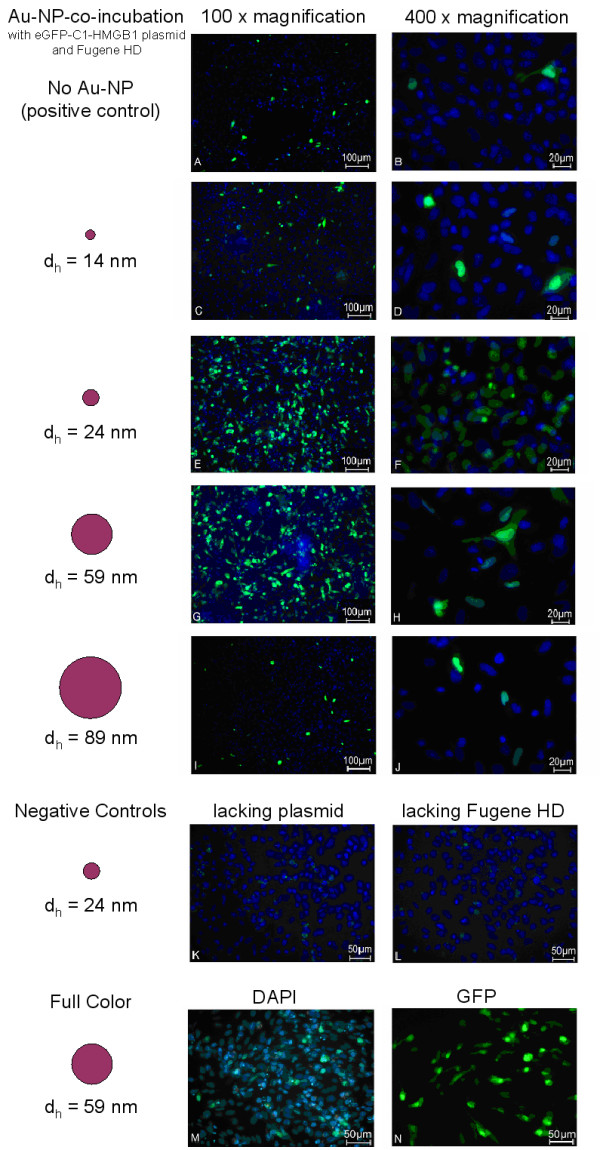
**The effect of co-transfecting plasmid DNA and laser generated gold nanoparticles on the bioactivity of GFP-HMGB1 fusion protein**. **Images A to I (vertical) show a 100 fold magnification and B to J (vertical) a 400 fold magnification**. Images A and B represent the positive control I: a transient transfection of MTH53a cells by Fugene HD reagent with the eGFP-C1-HMGB1 plasmid without Au-NP incubation. Cells in images C to J are treated like control I but include incubation of the plasmid with 14 nm sized Au-NP (C and D), 24 nm sized Au-NP (E and F), 59 nm sized nanoparticles (G and H) and 89 nm sized Au-NP (I and J), respectively. Image K and L represent the negative controls II and III. M and N are full color images of DAPI and GFP fluorescence.

Transfected cells were washed with PBS, fixed in a 4% paraformaldehyde/PBS solution (pH 7.5) for 30 min at room temperature and washed again with PBS. Afterwards, the cells were incubated with 10 μl of mounting medium containing DAPI (4',6-diamidino-2-phenylindole) for fluorescent visualization of nucleic DNA (Vecta Laboratories, Burlingame, USA). Fluorescence microscopy was performed using the Carl Zeiss Axioskop 2 and images were recorded with the Axiovision Software. eGFP fluorescence was measured employing wavelength filter set 10 (Carl Zeiss MicroImaging, Göttingen, Germany), while DAPI fluorescence was measured employing wavelength filter set 2 (Figure 2A to 2M). Both fluorescence images were taken with a Zeiss 2-channel Axiocam MRm camera. Both images were then merged in a single image. Full colour images were taken with a Zeiss Axiocam HRc (Figure 2M and 2N). The uptake of plasmid DNA (efficiency of transfection) was estimated taking into account the quantity of cells within an ocular's visual field. Thus the estimation was done comparing the number of cells showing green fluorescence protein expression (green staining) and cells showing blue DAPI fluorescence dye staining.

### Co-transfection of plasmid DNA and laser-generated gold nanoparticles

As the HMGB1 protein is a transcription factor, it binds strongly to nuclear DNA. We therefore may assume that cell nuclei containing strong eGFP fluorescence represent successful functional transfection events. All cells transfected with AuNP-incubated plasmid DNA showed strong colocalised eGFP and DAPI staining (Figure 2), whilst the negative controls, cells treated with Au-NP and FHD (AuNP of d_h _= 24 nm), showed no eGFP fluorescence (Figure 2K). We therefore conclude that co-incubation of AuNP with the plasmid DNA encoding the recombinant canine HMGB1 neither prevents the mediated uptake of the vector in presence of a transfection reagent nor has any visible effect on the transport and biological functionality of the synthesised fusion proteins.

By comparing fluorescence images of the cells co-incubated with the AuNPs of different sizes and to cells incubated without AuNPs, we were able to compare transfection efficiencies in each case. We estimate that the achieved efficiency of DNA transfection for the sample containing 14 nm AuNPs was approx. 15 ± 5% (Figure 2C and 2D).

The highest observed transfection efficiencies were achieved using 24 nm and 59 nm Au-NPs (50 ± 5% and 50 ± 10% respectively, see Figure 2E to 2H). The Au-NPs showed size dependent effects concerning the observed transfection efficiencies (see Table 1). Exemplarily, Au-NPs of a medium size (d_h_: 24 and 59 nm) showed the highest effects. Thus, the observed GFP fluorescence of the respective fusion proteins was so intense that it even leaked into the DAPI channel (Fig 2M and 2N respectively for AuNP of d_h _= 59 nm). Further negative control samples containing DNA- co-incubated AuNPs missing FHD, showed no recombinant protein expression, proving that our AuNPs did not act as transfection reagent themselves. (Figure 2L). The cell population seems to go along with transfection efficiency, as the observed seeding density was in all wells similar prior to transfection.

**Table 1:** Summary of estimated transfection efficiencies

**Size** **Au-NP (d_h_)**	**Estimated Transfection Efficiency (%)**	**Figure**
Positive controls	10 ± 2	A and B
14 nm	15 ± 5	C and D
24 nm	50 ± 5	E and F
59 nm	50 ± 10	G and H
89 nm	8 ± 3	I and J
Negative controls	-	K and L

Differently sized Au-NPs were incubated with plasmid DNA and transfected into the canine MTH53a cellline

### Shift assay

**Figure 3:**
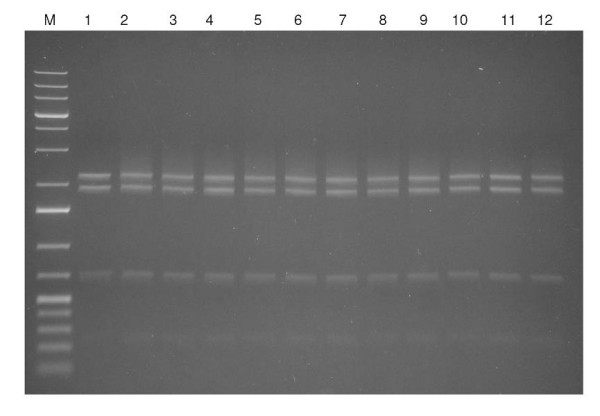
**Au-NPs/DNA and HMGB1 protein mobility shift assay**. **M**: GeneRuler 1 kb Plus (Fermentas), **lane 1**: 170 ng plasmid (NcoI digested); **lane 2**: 170 ng plasmid (NcoI digested) and 1.5 μg HMGB1; **lanes 3-6**: 170 ng plasmid 170 (NcoI digested) and 1.5 μg HMGB1 in 0.1 nM, 0.5 nM, 1.0 nM and 2.5 nM AuNPs suspensions, size d_h _24 nm; **lane 7**: 170 ng plasmid (NcoI) digested and 1.5 μg HMGB1 and 90 ng pure Au suspension, size d_h _24 nm; **lanes 8-11**: 170 ng plasmid (NcoI digested) and 1.5 μg HMGB1 in 0.1 nM, 0.5 nM, 1.0 nM and 2.5 nM AuNPs suspensions, size d_h _59 nm; **lane 12**: 170 ng plasmid (NcoI) digested and 1.5 μg HMGB1 and 50 ng pure Au suspension, size d_h _59 nm.

We performed binding experiments with plasmid DNA (eGFP-C1-HMGB1) and respective Au-NPs of different sizes and with various concentrations. We digested the co-incubated batches with a *NcoI *restriction enzyme (Fermentas, St Leon Rot, Germany) and separated the resulting DNA fragments in a 1.5% agarose gel. No significant shift alterations could be observed in the DNA mobility pattern. To ensure that this phenomenon is also valid in presence of proteins we added purified HMGB1 protein (Centre for Human Genetics, Bremen, Germany) to the batches. Akin to the DNA mobility pattern of digested Plasmid DNA and HMGB1 without Au-NPs (lane 2, Figure 3) we could not detect any significant change in the shift pattern (see lanes 3 to 12, Figure 3). Consequently the DNA/Au-NPs complexes serve as substrates for the DNA-bindig protein HMGB1.

## Conclusion

In conclusion, incubation of uncoated, positively charged AuNPs with a DNA plasmid that encodes recombinant eGFP-C1-HMGB1 fusion protein for 24 hours before cellular transfection does not seem to alter the protein expression and the protein functionality (DNA binding), while the presence of AuNPs seems to have a significantly positive effect on the transfection efficiencies. The observed effect was size-dependent: medium sized AuNPs enhanced transfection efficiency nearly 6 fold. These results support the hypothesis that laser-generated AuNPs present a good alternative to chemically synthesized nanoparticles and are especially suitable for biomedical applications.

## Competing interests

The authors declare that they have no competing interests.

## Authors' contributions

SP carried out the nanoparticle generation and partial drafting of the manuskript, JTS carried out the transfections, fluorescence microscopy analysis and partial drafting of the manuscript, SW performed cell culture and DNA preparation, AR generated the recombinant eGFP-C1-HMB1 plasmid, SB principal study design, manuscript drafting and supervision of nanoparticle work, HME principal design, partial manuscript drafting and supervision of molecular and cell biologic work. IN and JB participated in the conception design of the study. All authors read and approved the final manuscript.
